# Association of cumulative monocyte to high-density lipoprotein ratio with the risk of type 2 diabetes: a prospective cohort study

**DOI:** 10.1186/s12933-022-01701-7

**Published:** 2022-12-03

**Authors:** Dan Wu, Yulong Lan, Yuancheng Xu, Songna Xu, Yuejun Huang, Lois Balmer, Gehendra Maharra, Wencan Xu, Wei Wang, Shouling Wu

**Affiliations:** 1 The Second Affiliated Hospital of Shantou University Medical College, Shantou, 515041 China; 2 Department of Endocrinology, The First Affiliated Hospital of Shantou University Medical College, NO. 57, Changping Road, Jinping District, Shantou, 515041 Guangdong China; 3 Centre for Precision Health, School of Medical and Health Sciences, Edith Cowan University, Room 521, Building 21/270 Joondalup Drive, Perth, WA 6027 Australia; 4 Shantou University Medical College, Shantou, 515041 China; 5 Beijing Key Laboratory of Clinical Epidemiology, School of Public Health, Capital Medical University, Beijing, 100069 China; 6 School of Public Health, Shandong First Medical University & Shandong Academy of Medical Sciences, Tai’an, 271000 China; 7 Department of Urology, The University of Hong Kong-Shenzhen Hospital, Haiyuan Road, Shenzhen, 518053 China; 8 Department of Cardiology, Kailuan General Hospital, Xinghua East Road, Tangshan, 063000 China

**Keywords:** Type 2 diabetes, Cohort study, Monocyte to high-density lipoprotein ratio, Inflammation, Cumulative exposure

## Abstract

**Background:**

Recent studies have established that monocyte-derived inflammation plays a central role in the pathogenesis of type 2 diabetes mellitus (T2DM). It is unclear whether chronic metabolic inflammation, reflected by the cumulative monocyte to high-density lipoprotein ratio (CumMHR), predisposes the general population to T2DM.

**Methods:**

This study included 40,813 participants without diabetes from a real-life, community-based cohort (the Kailuan Study) attending a 2-year cycle of health survey since 2006. Cumulative exposure was obtained from 2006/2007 to 2010/2011. Follow-up started at 2010/2011 and through 2020. Multivariable-adjusted Cox regression models were used to calculate the CumMHR-associated risk of incident T2DM.

**Results:**

Over a median follow-up period of 7.98 (IQR: 5.74–8.87) years, 4,848 T2DM cases occurred. The CumMHR was positively associated with the risk of incident T2DM after adjusting for age, sex, smoking, drinking habits, physical activities, BMI, triglyceride-glycemia index, log(leukocyte count), log(hsCRP), blood pressure, renal function, and medication uses with adjusted HRs of 1.0 (ref.), 1.18 (1.05‒1.25), 1.17 (1.07‒1.27), 1.38 (1.26‒1.50), respectively, in CumMHR Quartiles 1, 2, 3 and 4. When follow-up ended at 2014/2015, the short-term (4‒year) adjusted T2DM risks in CumMHR Quartiles 2, 3, and 4 were 1.14 (1.01‒1.29), 1.17 (1.04‒1.32), 1.40 (1.25‒1.58), respectively, relative to Quartile 1. A significant interaction between CumMHR and cumulative high-sensitivity C-reactive protein (CumCRP) was observed (*P*-interaction: 0.0109). The diabetic risk in the highest quartile of CumMHR was higher (1.53 [1.28‒1.84]) when CumCRP < 1 mg/L, attenuated with increasing CumCRP levels (1 ~ 10 mg/L) and disappeared in CumCRP ≥ 10 mg/L. Hypertension, overweight, or smoking habits further modified the CumMHR-associated diabetic risk.

**Conclusions:**

Cumulative MHR may be a promising supplement to hsCRP for more comprehensively assessing the influence of metabolic inflammation on T2DM susceptibility. For primary prevention, targeting high CumMHR, especially in cases at low risk of diabetes defined by traditional risk factors, may further help reduce the diabetic risk.

**Supplementary Information:**

The online version contains supplementary material available at 10.1186/s12933-022-01701-7.

## Background

The inflammatory nature of type 2 diabetes mellitus (T2DM) is well established. Monocytes and their derived macrophages have been proposed to be highly engaged in the pathogenesis of T2DM [1], encompassing islet inflammation, beta-cell malfunction, and impaired insulin signaling [1–4]. Obesity, a well-known cause of T2DM, exhibits prominent monocytosis [5], which enhances insulin resistance (IR) through the infiltration of monocyte-derived macrophages into adipose tissue [6]. Animal studies have reported increased monocytosis in mouse models of diabetes [7, 8]. Despite a lack of specific monocyte data on human subjects with T2DM, observational studies have noted that increases in label-free leukocyte counts enhance the diabetic risk [9]. Multiple well-known risk factors (e.g., dietary habits [10], sleep disruptions [11], and chronic stress [12]) for chronic metabolic diseases are involved in increased monocytosis.

Importantly, lipid metabolism has a profound effect on hematopoiesis. High-density lipoprotein (HDL) has been identified to negatively mediate monocytosis [13] and attenuate adverse monocyte-derived proinflammatory effects by suppressing monocyte proliferation, activation and migration [13–15]. The imbalance of monocytes and HDL-C, i.e., the monocyte-to-HDL-C ratio (MHR), has been proposed to indicate low-grade metabolic inflammation [16]. The MHR was first found to elevate the risk of cardiovascular events among 340 patients with chronic kidney disease (CKD) in 2004 [16]. Results from later cohort studies supported that the MHR might be a novel marker of inflammation and potentially a prognostic indicator of CVD [16–18] and kidney diseases [19, 20]. Notably, one hallmark of frank diabetes is a low level of high-density lipoprotein cholesterol (HDL-C), resulting from the milieu of enriched triglycerides (TGs) and IR [21, 22]. As such, the high MHR may indicate a deteriorated proinflammatory status enhanced by elevated monocytosis and the specific HDL deficiency in diabetes-prone milieu, hence being a potential candidate for assessing the inflammatory risk in diabetes. However, data supporting an epidemiological link between the MHR and T2DM incidence have been limited thus far.

Statistical data have substantiated the concerning increasing trend of T2DM [23]. Identifying novel biomarkers is a promising way to improve the current risk prediction tools and offers new insights for the development of additional therapeutic targets of T2DM. In the present study, we took advantage of the longitudinal cohort design of the Kailuan Study and used long-term cumulative MHR to assess the chronic metabolic inflammatory exposure, aiding the application of MHR into community-based practice on T2DM prevention in the general population.

## Methods

### Study participants

The Kailuan Study, a large ongoing prospective real-life cohort study in Tangshan, North China, was initially designed to examine risk factors for chronic diseases (trial registration number: ChiCTR-TNC-11001489). Details of the study design are described elsewhere [24–27]. In brief, participants in this cohort have undergone biennial health surveys since 2006/2007. The latest health survey was administered on Dec. 31, 2021. Each participant provided written informed consent before enrollment. This current investigation was a subset analysis of the Kailuan Study, and it was approved by the Kailuan General Hospital Ethics Committee, China (2006‒2005) and the Human Research Ethics Committee of Edith Cowan University (2021‒03159‒BALMER).

**Fig. 1:**
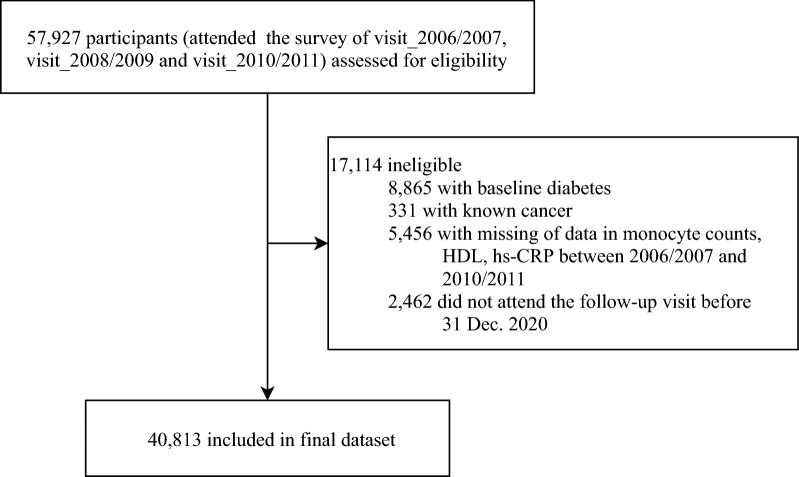
Flowchart of study participants

For the current study, the exposure period was from Visit_2006/2007 to Visit_2010/2011. The follow-up started from 2010/2011 through Dec. 31, 2020. Baseline description for the current study were determined according to the information at the commencement of follow-up. A flowchart of the participants in the current study is shown in Fig. 1. Among 57,927 participants who attended the first three health surveys between 2006/2007 and 2010/2011, we excluded those with diabetes (n = 8865) and known cancer (n = 331) at baseline, those with incomplete data on fasting blood glucose (FBG), monocyte counts, high-sensitivity C-reactive protein (hsCRP) levels, and HDL-C during the exposure period (n = 5456). We also excluded participants who did not attend any one of the follow-up health visits with FBG tests (n = 2462). A total of 40,813 participants were included in the final analysis. The number of participants and participation in the follow-up visits are reported in Additional file 1: Table S1.

### Exposure

Cumulative exposure was assessed in an approximately 4‒year (median 3.9, interquartile range [IQR]: 3.7‒4.2) period. The cumulative MHR (CumMHR) was calculated as [(MHR_Visit1 + MHR_Visit2)/2 × (date_Visit2 - date_Visit1)] + [(MHR_Visit2 + MHR_Visit3)/2 × (date_Visit3 - date_Visit2)] [28–30], where the MHR = the monocyte count/HDL-C. The cumulative monocyte count (CumMON), HDL-C (CumHDL-C), TyG (CumTyG) and high-sensitivity C-reactive protein level (CumCRP) were also calculated using the same algorithm. Subgroup analyses were performed according to the CumMHR, CumMON, and CumHDL-C quartiles.

### Ascertainment of outcome

The primary endpoint of this study was the incidence of T2DM (International Classification of Diseases, 10^th^ revision [ICD-10]: E11). Participants with FBG ≥ 7.0 mmol/L, a self-reported diagnosis by a physician, or a self-reported use of oral glucose-lowering medications with or without insulin use were considered to have T2DM [31, 32]. Confirmation of participant mortality was performed with reference to the information from local government vital statistics offices [33]. The date of diabetes onset was defined as the first follow-up examination at which a participant met the diagnostic criteria. The follow-up ended at the date of diabetes onset, death, or the last follow-up before Dec. 31, 2020, whichever came first. The secondary endpoint was the onset of impaired fasting glucose (IFG, defined as FBG levels ≥ 6.1 mmol/L [34]) among those without IFG during the exposure period.

### Covariates

Sociodemographic, lifestyle, medical, and medication history variables were collected via face-to-face interviews using a standard questionnaire, as detailed elsewhere [24]. Anthropometrics, including participant height, weight, and blood pressure, were assessed by trained physicians in accordance with standardized protocols. Laboratory assays encompassing routine blood tests (including leukocyte and monocyte counts), FBG, low-density lipoprotein cholesterol (LDL-C), TGs, total cholesterol (TC), and HDL-C as well as levels of creatinine, and hsCRP were measured at the central laboratory in Kailuan General Hospital using a Hitachi 7600 autoanalyzer (Hitachi; Tokyo, Japan). As there were no available insulin data for calculating the HOMA-IR as an assessment of insulin resistance (IR), we used the triglyceride-glycemia index (TyG) (calculated as ln [fasting triglycerides (mg/dL) × fasting plasma glucose (mg/dL)/2] [35] and ultrasonography-measured fatty liver degrees (normal, gentle, moderate and severe [36]) as alternative assessments for IR [37]. The estimated glomerular filtration rate (eGFR) was calculated according to the Chronic Kidney Disease Epidemiology Collaboration Creatinine Equation [38]. Hypertension (ICD-10: I10) was defined as diastolic blood pressure (DBP) ≥ 90 mmHg, systolic blood pressure (SBP) ≥ 140 mmHg, self-reported use of antihypertensive drugs, or self-reported a prior hypertension diagnosis. Blood pressure was categorized as follows: normal blood pressure (SBP < 140 mm Hg and DBP < 90 mm Hg), grade I hypertension (140 ≤ SBP < 160 mm Hg or 90 ≤ DBP < 100 mm Hg), grade II hypertension (160 ≤ SBP < 180 mm Hg or 100 ≤ DBP < 110 mm Hg), and grade III hypertension (SBP ≥ 180 mm Hg or DBP ≥ 110 mm Hg). BMI was calculated as weight (kg) divided by height squared (m^2^) and participants were categorized as underweight (BMI ≤ 18 kg/m^2^), normal weight (18 ≤ BMI < 25 kg/m^2^), overweight (25 ≤ BMI < 30 kg/m^2^), or obese (BMI ≥ 30 kg/m^2^). Participant smoking status was divided into three categories: never, former, and current. Participant alcohol consumption was categorized as “yes” or “no”.

### Statistical analysis

All data processing and statistical analysis were performed using SAS version 9.4 (SAS Institute, Cary, NC, USA). Missing values (< 2%) of potential covariates were imputed by multivariate chained imputation. We checked the normality of data distributions using the Kolmogorov–Smirnov test. For baseline descriptions, we present the mean ± standard deviation (SD), median with IQR, or number and percentage (%), as appropriate. To compare participants’ baseline characteristics across CumMHR quartiles, we used one-way analyses of variance (ANOVAs) or Kruskal–Wallis tests for continuous variables and Pearson chi-square tests for categorical variables.

The incidence rates (per 1000 person-years) of T2DM were calculated. Kaplan–Meier plots were generated with the log-rank test to compare the cumulative incidence. After confirming that the proportional hazards assumption was satisfied, Cox proportional hazard regression models were used to compare the hazard ratios (HRs) with 95% confidence intervals (CIs) for T2DM across the CumMHR subgroups. In addition, to determine the association of the CumMHR with short-term and long-term risks for diabetes, the datasets were divided into two subsets, i.e., diabetes occurred between 2010/2011 and 2014/2015 and diabetes occurred between 2014/2015 and December 2020. Interactions between the CumMHR and CumCRP, sex, age, and hypertensive and overweight statuses as well as smoking, drinking habits, physical activities and eGFR levels were tested by the likelihood test with the fully adjusted Cox proportional hazard regression model. Stratified analyses were performed when a significant interaction was observed. The multivariable-adjusted models were as follows: Model 1 adjusted for age (continuous), sex, smoking status, alcohol consumption, physical activity, education level, and BMI (categorical); Model 2 further adjusted for baseline FBG (continuous), eGFR (categorical), log(hsCRP), log(leukocyte), blood pressure category, dyslipidemia (yes or no), taking antihypertensive medication (yes or no), and taking lipid-lowering agents (yes or no); Model 3: additional adjustment for fatty liver degrees (normal, gentle, moderate and severe) on the basis of Model 2. Model 4: additionally adjusted for TyG instead of FBG in Model 2. Since MHR per se is actually a ratio reflects an interaction term in a statistical model, we further additionally adjusted for both of the separate components in an additional model (Model 5) to test the interaction term. Additionally, to address the main impacts of both components on CumMHR-associated T2DM risks, the associations between separate CumMON (Model 6) or CumHDL (Model 7) and incident T2DM were also examined.

In addition, to examine the robustness and consistency of our findings, several sensitivity analyses were performed with the Cox regression models. First, to address potential reverse causation, the study endpoints recorded at the first follow-up visit were excluded. Second, to minimize the influence of acute infection, participants with suspected acute infection (whichever hsCRP level ≥ 10 mg/L in the exposure period [39]) were excluded. Third, participants who took statins were excluded to address the potential confounding effect of statins on the study endpoint. Fourth, to minimize the influence of CVD, participants with preexisting CVD were excluded. Fifth, examined the CumMHR-associated T2DM risks after additional adjustment for cumulative TyG instead of baseline TyG as an assessment for cumulative IR (Model 8). Sixth, examine the role of CumMHR in incident T2DM after additional adjustment for cumulative hsCRP instead of baseline hsCRP (Model 9). Seventh, the analysis was repeated by extending the sample size among participants who underwent at least two MON and HDL-C measures in the exposure visits and imputing the missing data with the values measured in the nearest health examination.

Two-tailed *P* < 0.05 was considered statistically significant, except in the interaction analysis, where *P* < 0.1 was considered significant.

## Results

Baseline characteristics were determined according to the information provided at the start of follow-up (Table 1). The study participants had a mean age of 52.2 ± 11.8 years at baseline, and 75.1% of participants were men. Participants in higher CumMHR quartiles had higher CumCRP, CumTyG and baseline hsCRP, TyG levels, leukocyte counts, blood pressures (SBP and DBP), TGs, BMI, had more current drinkers and current smokers, meanwhile had lower levels of TC, LDL-C, HDL-C, eGFR and education. Notably, there was no significant difference in baseline FBG across the CumMHR quartiles (*P* = 0.0921) or differences in the baseline use of statins, fibrates, and antihypertensives (Table 2).

**Table 1:** Baseline characteristics

Characteristic	Total (n = 40,813)	CumMHR < 0.172 (n = 10,203)	0.172 ≤ CumMHR < 0.234 (n = 10,203)	0.234 ≤ CumMHR < 0.316 (n = 10,203)	CumMHR ≥ 0.316 (n = 10,204)	*P*-value
Age, years	52.2 ± 11.8	53.5 ± 12.0	52.9 ± 11.9	51.9 ± 11.7	50.6 ± 11.5	< 0.0001
Male, n (%)	30,634 (75.1)	6174 (60.5)	7515 (73.7)	8192 (80.3)	8753 (85.8)	< 0.0001
CumCRP, mg/L	1.6 (0.8‒3.1)	1.2 (0.6‒2.3)	1.5 (0.8‒2.9)	1.7 (0.9‒3.4)	2.0 (1.1‒4.0)	< 0.0001
CumMON, 10^9^/L	0.3 (0.3‒0.4)	0.2 (0.2‒0.3)	0.3 (0.3‒0.3)	0.4 (0.3‒0.4)	0.5 (0.4‒0.6)	< 0.0001
CumHDL‒C, mmol/L	1.5 (1.3‒1.8)	1.7 (1.5‒2.0)	1.6 (1.4‒1.8)	1.5 (1.3‒1.6)	1.4 (1.2‒1.6)	< 0.0001
CumTyG	8.9 ± 1.1	8.5 ± 1.1	8.8 ± 1.1	9.0 ± 1.1	9.3 ± 1.1	< 0.0001
BMI, kg/m^2^	25.0 ± 3.3	24.2 ± 3.2	24.8 ± 3.3	25.3 ± 3.3	25.6 ± 3.4	< 0.0001
*BMI categorical*						< 0.0001
Underweight	531 (1.3)	225 (2.2)	137 (1.3)	87 (0.9)	82 (0.8)	
Normal weight	20,599 (50.5)	6130 (60.1)	5353 (52.5)	4807 (47.1)	4309 (42.2)	
Overweight	16,774 (41.1)	3414 (33.5)	4046 (40.0)	4498 (44.1)	4816 (47.2)	
Obesity	2909 (7.1)	434 (4.3)	667 (6.5)	811 (8.0)	997 (9.8)	
SBP, mm Hg	129.6 ± 18.6	128.0 ± 18.8	129.6 ± 18.7	130.2 ± 18.6	130.7 ± 18.1	< 0.0001
DBP, mm Hg	80.7 (79.3‒90.0)	80.0 (77.7‒90.0)	80.7 (80.0‒90.0)	81.7 (80.0‒90.0)	83.0 (80.0‒90.0)	< 0.0001
FBG, mmol/L	5.2 ± 0.6	5.2 ± 0.6	5.2 ± 0.6	5.2 ± 0.6	5.2 ± 0.6	0.0921
HDL-C, mmol/L	1.5 (1.2‒1.8)	1.8 (1.5‒2.1)	1.6 (1.3‒1.9)	1.4 (1.2‒1.7)	1.3 (1.1‒1.5)	< 0.0001
LDL-C, mmol/L	2.6 ± 0.8	2.6 ± 0.8	2.6 ± 0.8	2.6 ± 0.8	2.5 ± 0.8	< 0.0001
TC, mmol/L	5.0 ± 1.0	5.2 ± 1.0	5.0 ± 1.0	4.9 ± 0.9	4.8 ± 0.9	< 0.0001
TG, mmol/L	1.3 (0.9‒1.8)	1.1 (0.8‒1.6)	1.2 (0.9‒1.7)	1.3 (0.9‒1.9)	1.4 (1.1‒2.1)	< 0.0001
TyG	8.6 ± 0.6	8.5 ± 0.6	8.6 ± 0.6	8.7 ± 0.6	8.7 ± 0.6	< 0.0001
Leukocyte counts, 10^9^/L	6.1 (5.2‒7.2)	5.3 (4.5‒6.2)	5.9 (5.1‒6.9)	6.4 (5.5‒7.4)	7.0 (6.0‒8.2)	< 0.0001
eGFR, ml/min/1.73m^2^	81.9 (59.2‒96.8)	90.0 (68.7‒100.6)	83.6 (60.2‒97.5)	74.5 (57.0‒94.7)	69.4 (55.9‒93.0)	< 0.0001
HsCRP, mg/L	1.0 (0.5‒2.4)	0.9 (0.5‒1.8)	1.0 (0.5‒2.2)	1.1 (0.5‒2.6)	1.3 (0.5‒3.2)	< 0.0001
*Alcohol consumption, n (%)*						< 0.0001
No	26,752 (65.5)	6966 (68.3)	6703 (65.7)	6582 (64.5)	6501 (63.7)	
Yes	14,061 (34.5)	3237 (31.7)	3500 (34.3)	3621 (35.5)	3703 (36.3)	
*Smoking status, n (%)*						< 0.0001
Never smoker	25,248 (61.9)	7235 (70.9)	6447 (63.2)	6082 (59.6)	5484 (53.7)	
Former smoker	1796 (4.4)	382 (3.7)	469 (4.6)	451 (4.4)	494 (4.8)	
Current smoker	13,769 (33.7)	2586 (25.3)	3287 (32.2)	3670 (36.0)	4226 (41.4)	
Family history of diabetes	2162 (5.3)	541 (5.3)	511 (5.0)	511 (5.0)	599 (5.9)	0.0179
*Education, n (%)*						< 0.0001
Less than high school	31,295 (76.7)	7329 (71.8)	7773 (76.2)	8019 (78.6)	8174 (80.1)	
High school and above	9518 (23.3)	2874 (28.2)	2430 (23.8)	2184 (21.4)	2030 (19.9)	
*Physical activities, n (%)*						< 0.0001
Low	13,749 (33.7)	3911 (38.3)	3503 (34.3)	3276 (32.1)	3059 (30.0)	
Moderate	21,329 (52.3)	4492 (44.0)	5217 (51.1)	5628 (55.2)	5992 (58.7)	
High	5735 (14.1)	1800 (17.6)	1483 (14.5)	1299 (12.7)	1153 (11.3)	
Hypertension	19,733 (48.3)	4373 (42.9)	4865 (47.7)	5101 (50.0)	5394 (52.9)	< 0.0001
Dyslipidemia	11,100 (27.2)	2363 (23.2)	2462 (24.1)	2826 (27.7)	3449 (33.8)	< 0.0001
*Fatty liver degree*						< 0.0001
Normal	24,763 (60.7)	7030 (68.9)	6362 (62.4)	5852 (57.4)	5519 (54.1)	
Gentle	10,535 (25.8)	2312 (22.7)	2627 (25.8)	2789 (27.3)	2807 (27.5)	
Moderate	4657 (11.4)	795 (7.8)	1052 (10.3)	1312 (12.9)	1498 (14.7)	
Severe	858 (2.1)	66 (0.7)	162 (1.6)	250 (2.5)	380 (3.7)	
Antihypertensives, n (%)	2201 (5.4)	593 (5.8)	551 (5.4)	537 (5.3)	520 (5.1)	0.1302
Statin, n (%)	230 (0.6)	56 (0.5)	62 (0.6)	45 (0.4)	67 (0.7)	0.1949
Fibrate, n (%)	65 (0.2)	9 (0.1)	16 (0.2)	17 (0.2)	23 (0.2)	0.1075

CumMHR: cumulative monocyte to high-density lipoprotein cholesterol ratio; CumHDL-C: cumulative high-density lipoprotein cholesterol; CumMON: cumulative monocytes; CumTyG: cumulative triglyceride-glycemia index; BMI: body mass index, SBP: systolic blood pressure, DBP: diastolic blood pressure, FBG: fasting blood glucose; TC: total cholesterol; TG: triglyceride; TyG: triglyceride-glycemia index; LDL-C: Low-density lipoprotein cholesterol, HDL-C: high-density lipoprotein cholesterol; eGFR: estimated glomerular filtration rate; HsCRP: hypersensitive C-reactive protein

**Table 2:** CumMHR-associated type 2 diabetes risk

	CumMHR, HRs (95% CIs)				*P* for trend	Per SD
	Quartile1	Quartile 2	Quartile 3	Quartile 4		
*Entire cohort*						
Event/Total	890/10203	1140/10203	1253/10203	1565/10204		
Incidence rate	12.12	15.68	17.52	22.38		
Unadjusted model	Reference	1.29 (1.19,1.41)	1.44 (1.32,1.57)	1.83 (1.69,1.99)	< 0.0001	1.24 (1.20,1.27)
Model 1	Reference	1.20 (1.10,1.31)	1.29 (1.18,1.41)	1.59 (1.46,1.74)	< 0.0001	1.18 (1.14,1.21)
Model 2	Reference	1.18 (1.08,1.29)	1.24 (1.13,1.36)	1.50 (1.35,1.65)	< 0.0001	1.16 (1.12,1.19)
Model 3	Reference	1.17 (1.07,1.27)	1.21 (1.10,1.32)	1.46 (1.33,1.60)	< 0.0001	1.15 (1.11,1.19)
Model 4	Reference	1.18 (1.05,1.25)	1.17 (1.07,1.27)	1.38 (1.26,1.50)	< 0.0001	1.11 (1.08,1.15)
Model 5	Reference	1.18 (1.06,1.31)	1.21 (1.06,1.38)	1.44 (1.21,1.72)	< 0.0001	1.24 (1.11,1.39)
Model 6	Reference	1.08 (0.98,1.19)	1.05 (0.94,1.17)	1.16 (1.02,1.34)	< 0.0001	1.01 (0.95,1.08)
Model 7	Reference	1.16 (1.06,1.27)	1.18 (1.08,1.30)	1.40 (1.27,1.55)	< 0.0001	1.12 (1.08,1.16)
Model 8	Reference	1.14 (1.04,1.25)	1.17 (1.07,1.28)	1.40 (1.27,1.53)	< 0.0001	1.13 (1.09,1.17)
Model 9	Reference	1.12 (1.03,1.23)	1.13 (1.03,1.23)	1.29 (1.18.1.41)	< 0.0001	1.08 (1.05,1,12)

Model 1: adjusted for age, sex, education, smoke, drinking status, physical activities, family history of diabetes, BMI; Model 2: Model 1 + FBG, blood pressure, eGFR, dyslipidemia (yes or no), antihypertensives (yes or no), lipid-lowering drugs (yes or no), log(hsCRP),log(leukocyte count); Model 3: Model 3 + fatty liver degree (categorical); Model 4: Model 2 + TyG instead of FBG; Model 5: Model 4 + log (CumHDL) + log (CumMON); Model 6: Model 3 + log (CumMON); Model 7: Model 3 + log (CumHDL-C); Model 8: Model 4 + log(CumCRP) instead of log(hsCRP); Model 9:Model 4 + CumTyG instead of TyG

The incidence rate is per 1,000 person-years

Per SD: risk per unit increment in CumMHR (0.1995)

Abbreviations as Table 1

**Fig. 2:**
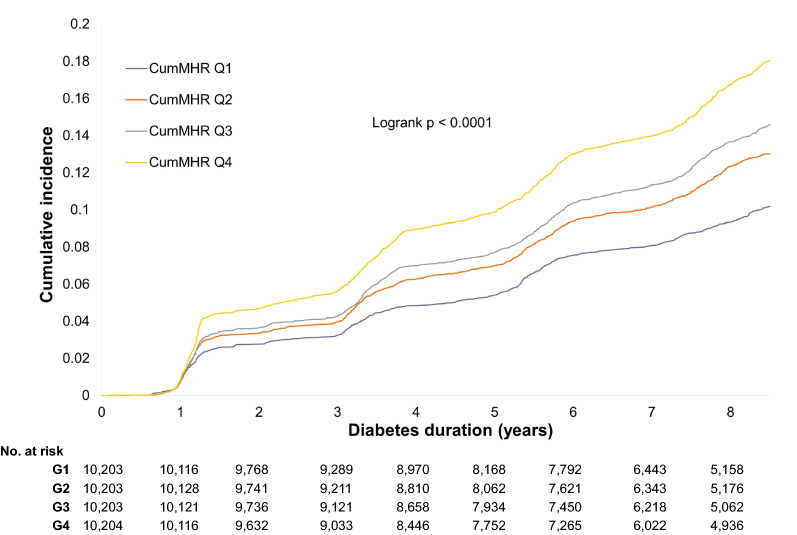
Kaplan‒Meier curves of cumulative incidence of T2DM. CumMHR Quartile 1 was used as the Reference group. CumMHR: cumulative monocytes to high-density lipoprotein ratio

Over a medium follow-up of 7.98 (IQR: 5.74‒8.87) years, 4848 cases of T2DM were documented among the 40,813 study participants. There was a graded increase in incidence rates across CumMHR quartiles. The HRs (95% CIs) in CumMHR Quartile 2, 3 and 4 were 1.18 (1.08‒1.29), 1.24 (1.13‒1.36) and 1.50 (1.35‒1.65), respectively, when compared to that of Quartile 1 (*P*-trend < 0.0001) after adjusting for age, sex, BMI, smoking, drinking habits, physical activities, family history of diabetes, blood pressure, FBG, eGFR, dyslipidemia, log(leucocyte count) and log(hsCRP) values. Further adjustment for insulin resistance (IR) attenuated the associations, with aHRs (95% CIs) in CumMHR Quartiles 2, 3 and 4 of 1.17 (1.07‒1.27), 1.21 (1.10‒1.32) and 1.46 (1.33‒1.60), respectively, after adjustment for fatty liver degrees (Model 3), and 1.18 (1.05‒1.25), 1.17 (1.07‒1.27), and 1.38 (1.26‒1.50), respectively, after adjustment for TyG levels (Model 4)]. Notably, the CumMHR-associated T2DM risks were more prominent after adjustment for both components [log(CumMON) and log(CumHDL)] (Model 5). The CumMHR-associated risks appeared to be greatly attenuated after additional adjustment for sperate log(CumMON) (Model 6) in comparison to additional adjustment for log(CumHDL) (Model 7). Furthermore, the CumMHR-diabetes association was somewhat attenuated but still significant after additional adjustment for cumulative hsCRP (Model 8) and TyG levels (Model 9). Figure 2 displays the Kaplan–Meier curves of the cumulative incidence of T2DM. Additionally, when the follow-up ended in 2014/2015, the short-term (median: 4.11 [IQR:3.36‒4.42] years) HRs (95% CIs) for incident T2DM in CumMHR Quartiles 2, 3, and 4 were 1.14 (1.01‒1.29), 1.17 (1.04‒1.32), and 1.40 (1.25‒1.58), respectively, relative to Quartile 1, with a 1.13 (1.08‒1.17) increase in risk per 1-SD increase in log(CumMHR) (*P*-trend: < 0.001). In terms of the long-term risk (excluding diabetes occurring within the first two follow-up visits), the HRs (95% CIs) for incident T2DM across the increasing CumMHR quartiles (Quartiles 1, 2, 3, and 4) were 1,0 (ref.), 1.16 (1.02,1.32), 1.16 (1.02‒1.33),1.34 (1.18‒1.53), respectively, with a 1.11 (1.06‒1.16) increase in risk per 1‒SD increase in log(CumMHR) (*P*-trend < 0.001; Additional file 1: Table S2).

We further evaluated the association between the CumMHR and the incidence of T2DM in 7091 participants who exhibited IFG at any visit during the exposure period and documented 2166 cases of diabetes during the follow-up. The CumMHR was independently associated with the risk of T2DM, with an adjusted HR (95% CI) of 1.58 (1.39‒1.79) in the highest CumMHR quartile. In participants without IFG (n = 33,722), 2682 cases of T2DM occurred. The highest CumMHR quartile had a significant risk of T2DM (HR: 1.41, 95% CI [1.25‒1.58], Additional file 1: Table S3). No significant interaction between IFG status and CumMHR was found to be associated with developing T2DM (*P*-interaction: 0.7130). We additionally examined the CumMHR-associated risks for incident IFG among participants free of IFG during the exposure period. A total of 8293 participants had IFG onset among the 33,722 non-IFG participants. Cumulative MHR was significantly associated with IFG onset independent potential risk factors (Additional file 1: Table S4).

**Fig. 3:**
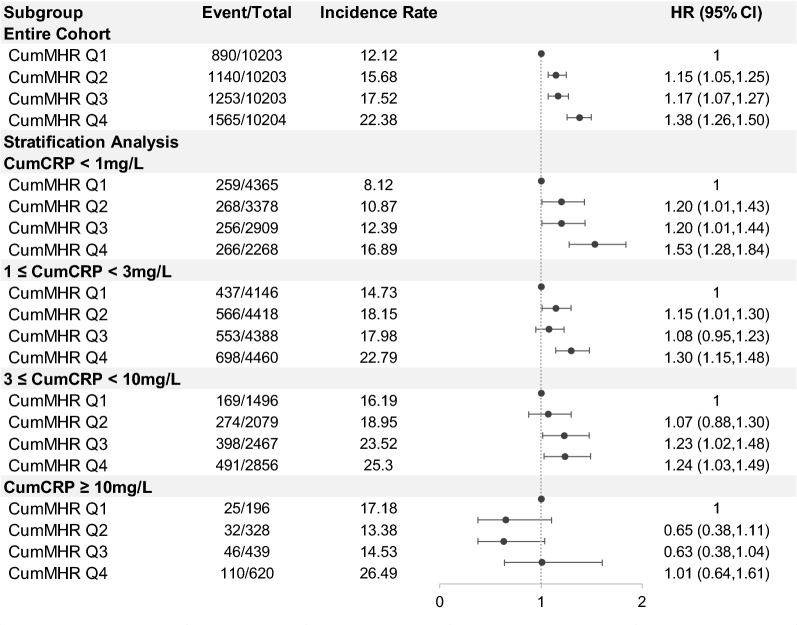
Forest plot of multivariable-adjusted association between CumMHR and T2DM onset in the overall participants and stratified by CumCRP levels. *P*-interaction: CumMHR quartiles × CumCRP thresholds (1, 3, 10 mg/L) = 0.0109. All models are adjusted for age, sex, education, smoking, drinking status, physical activities, family history of diabetes, BMI (categorical), blood pressure (categorical), eGFR (categorical), dyslipidemia (yes or no), antihypertensives (yes or no), lipid-lowering drugs (yes or no), TyG (continuous), log(leukocyte counts)(continuous), log(hsCRP) (limited to in the entire cohort) BMI: body mass index; CumCRP: cumulative hypersensitive C-reactive protein; CumMHR: cumulative monocyte to high-density lipoprotein cholesterol ratio, eGFR: estimated glomerular filtration rate; TyG: triglyceride-glycemia index

Notably, a significant interaction between the CumMHR and CumCRP was observed and associated with incident T2DM [*P*-interaction: CumMHR quartiles × CumCRP thresholds (1, 3, 10 mg/L) = 0.0109]. Although the diabetic incidence rates tended to consistently increase with increases in CumCRP, the CumMHR-associated diabetic risks attenuated greatly with grade elevation in CumCRP levels, with adjusted HRs (95% CIs) of 1.53 (1.28‒1.84), 1.30 (1.15‒1.48), 1.24 (1.03‒1.49), and 1.01 (0.64‒1.61), respectively, for the CumCRP < 1, 1 ≤ CumCRP < 3, 3 ≤ CumCRP < 10, and CumCRP ≥ 10 mg/L strata (Fig. 3, Additional file 1: Table S5). In addition, significant interactions were observed between the CumMHR and participants’ hypertensive (*P*-interaction: 0.0300) and overweight statuses (*P*-interaction: 0.0885 for IR assessed by fatty liver degrees and 0.1481 for IR assessed by TyG) as well as smoking habits (never smokers, ex-smokers and current smokers) (*P*-interaction: 0.0805) (Additional file 1: Tables S6, S7, S8). As anticipated, the MHR-associated diabetic risks were attenuated by baseline hypertension, overweight, or smoking habits. The CumMHR-associated T2DM risks appeared to be more prominent among those with impaired renal function (eGFR < 90 ml/min/1.73m^2^), albeit an insignificant interaction among CumMHR and eGFR levels being found (*P*-interaction: 0.3725; Additional file 1: Table S9). There were no significant interactions between the CumMHR quartiles and sex (*P*-interaction: 0.2677), age subgroups (< 45, 45–60, ≥ 60 years) (*P*-interaction: 0.7306), alcohol consumption (yes or no) (*P*-interaction: 0.2942), physical activities (low, moderate, high) (*P*-interaction: 0.5122).

The results were robust, as indicated by the similar main outcomes in the sensitivity analyses when excluding events that occurred at the first follow-up visit, participants with suspected acute infections (hsCRP levels ≥ 10 mg/L during the visits in the exposure period), participants who took statins, who had baseline CVD, or who had missing data on the key covariates (Additional file 1: Table S10). Repeated analyses among participants with at least two MHR data in the exposure visits yielded similar results (Additional file 1: Table S11).

We additionally examined the associations between separate components of MHR (MON and HDL-C) and incident T2DM. Isolated cumulative monocyte counts influenced the adjusted HR (95% CI) when comparing the two extreme quartiles, at 1.33 (95% CI, 1.22‒1.45; *P*-trend < 0.001; Additional file 1: Table S12). Although a downward trend in the incidence rates and risk of T2DM in the crude model was observed with increasing CumHDL-C, there was not a statistically significant association between CumHDL-C and the risk of T2DM in the fully multivariable-adjusted model with IR assessed as fatty liver degree. However, higher HDL-C tended to be a risk factor for incident T2DM after adjusting for TyG (Additional file 1: Table S13).

Furthermore, we also investigated the association between the baseline MHR (BasMHR) and incident T2DM, with adjusted HRs (95% CIs) of 0.99 (0.91‒1.08), 1.17 (1.07‒1.28), and 1.22 (1.11‒1.33), respectively, in BasMHR Quartiles 2, 3, and 4, by reference to Quartile 1 (*P*-trend < 0.0001; Additional file 1: Table S14).

## Discussion

### Principle findings

For the first time, we reported that both the cumulative increase in a leukocyte subpopulation rich in monocytes and the MHR were independently associated with the risk of T2DM in the general population of 40,813 participants. Within a follow-up period of approximately eight years. More importantly, the CumMHR-associated risk for incident T2DM varied remarkably across different CumCRP strata and was particularly higher in the absence of elevated hsCRP levels. Additionally, hypertensive and overweight statuses as well as smoking preference further refined the MHR-associated diabetic risks.

### Strengths and limitations compared with other studies

Consistent with our findings, recent experiment-based studies have substantiated the critical role of monocyte-derived immunity, which contributes to beta-cell malfunction, insufficient insulin secretion and IR, in the pathogenesis of T2DM [1–4]. Glucose metabolism was found to impact monocyte activity [40]; treatment of hyperglycemia significantly reduced the number of circulating monocytes [41]. The prominent monocytosis observed in mouse models of obesity provided a source for the infiltration of macrophages into adipose tissue, driving the progression to IR and the onset of diabetes [6]. In line with these findings, increased monocytosis due to the activation of precursors in bone marrow has been observed in mouse models of diabetes [7, 8]. Additionally, reduced monocyte mobilization from the bone marrow after fasting [10] was significantly linked to the anti-inflammatory benefits of caloric restriction [42]. Indeed, reducing the number of circulating monocytes and macrophages may serve as a novel approach to reduce diabetes-related vascular complications [14, 40].

It is essential to know that the circulating monocyte pool is highly dynamic and substantially influenced by defective cholesterol metabolism. While LDL-C promotes monocytosis [43], HDL-C has the opposite effect [41]. Studies in recent years have continuously found that HDL-C has potent anti-inflammatory properties, particularly against monocytic inflammation [13–15]. Elevated levels of HDL-C decrease monocyte counts [13] by suppressing monocyte proliferation and motivation, as well as the proliferation of bone marrow progenitors with mechanisms involved favoring cholesterol efflux from these cells [14]. Notably, in IR and overt diabetes, low levels of HDL-C [21, 22] and impaired anti-inflammatory properties of HDL-C [44] are commonly observed. Thus, decreases in the suppression of monocyte-associated inflammation by HDL-C consequently aggravate the imbalance between pro- and anti-inflammatory processes, supporting the potential use of MHR as an appropriate marker for metabolic inflammation in diabetes.

Studies in recent years have increasingly yielded out the significance of MHR as a promising inflammation marker for cardiovascular diseases and kidney function from the predictive and prognostic perspectives, albeit in studies with relatively small sample sizes and short follow-up periods and were restricted to a specific group of patients. The results from our study fill in the gap in the current understanding of the relationships between the risk of T2DM and monocytes and the MHR, particularly on a large population scale.

Importantly, the attenuation of the positive graded association between quartiles of CumMHR and incident diabetes by increasing CumCRP levels is of interest. The risk upon exposure to the elevated CumMHR was higher in the absence of elevated CumCRP (CumCRP < 1 mg/L), attenuated with a gradual increase in CumCRP, and finally disappeared in the high-grade inflammation stratum (CumCRP ≥ 10 mg/L). Nevertheless, the incidence rates tended to consistently rise as CumCRP increased, consistent with the dose-dependent relationship between hsCRP levels and the risk of T2DM observed in previous studies [45, 46]. The significant CumMHR-CumCRP-interaction may be accounted for by the bidirectional relationship between hsCRP and monocytic inflammation in the biological background [47, 48]. On the one hand, the production of CRP is largely dependent on response to monocytic cytokines; elevated levels of monocytic interleukin-1β (IL-1β) and the IL-1β-secondary IL-6 drive the production of CRP [49, 50]. On the other hand, elevated CRP levels negatively mediate the activation and secretion of IL-1β [48] and subsequent production of monocytes [6], thereby attenuating monocyte-derived inflammation and protecting against detriments to overall health. As such, the downward trend in the CumMHR-associated diabetic risk that accompanied the increase in CumCRP levels is likely to result from the potential negative mediation of hsCRP on monocytic inflammation and the main influence of CRP rather than MHR on developing T2DM among participants with elevated CumCRP levels. In light of the evidence here, it is reasonable to postulate that the MHR may represent the earliest stage of metabolic inflammation, prior to the production of hsCRP and even prior to the elevation of circulating monocytic cytokines.

Furthermore, the presence of hypertension and overweight attenuated the CumMHR-associated diabetic risk. However, the incidence rate of diabetes increased in those with hypertension or overweight or smoking preference, consistent with them being risk factors for T2DM. Additionally, the subset analysis among non-CVD participants observed a moderately increased association between CumMHR and incident diabetes. These findings suggested that additional targeted assessment and management of CumMHR, especially in participants at low risk of diabetes defined by traditional risk factors, are instrumental for further reduction in the occurrence of diabetes.

### Implications of this study

The dual advantages of wide availability and cost-effectiveness of MHR in clinical settings warrant further attention into the potential use of MHR for determining the inflammatory risk for T2DM beyond that indicated by hsCRP levels. Measurements of cumulative MHR along with hsCRP levels may allow a more comprehensive assessment of the influence of chronic systemic metabolic inflammation on developing T2DM. Additionally, targeted assessment and management of CumMHR, especially in participants at low diabetic risks defined by traditional risk factors (e.g., CRP, hypertension, overweight, smoking habits), are instrumental for further improvement of diabetic outcome. Moreover, the significant epidemiological interaction between the cumulative MHR and hsCRP observed in this current study may provide directions for further in-depth exploration to disentangle the intertwined association of monocyte proliferation, HDL-C, and CRP levels in chronic metabolic inflammatory diseases.

China has the largest number of patients with diabetes worldwide, accounting for 24% of all patients with diabetes [51]. Given that prevention and remission of T2DM represent unmet, high-priority targets to alleviate the health and economic burden [51], it is essential to identify potential risk factors and promote primary, even primordial prevention against its occurrence. Convincing evidence has demonstrated that reduced monocytosis improves cardiovascular outcomes without compromising the emergency mobilization of monocytes for tissue repair and acute infectious inflammation, highlighting its compelling benefits [10]. Multiple manipulations, e.g., caloric restriction [10], a high-quality or sufficient sleeping manner [11], and moderate emotional and stress release [12], can promote reductions in circulating monocytes and elevations in HDL-C, thereby favoring both metabolic and cardiometabolic health.

To put our work in a broader context, the dual advantages of cost-effectiveness and wide availability of methods to determine the MHR in current clinical practice potentiate their widespread use as convenient tools for evaluating the risk of cardiometabolic diseases. Apart from the current T2DM disease, MHR was identified to be associated with renal disease onset among participants with or without diabetes [19, 52]. Higher monocyte counts and lower plasma high-density lipoprotein cholesterol levels were indicated in subjects with lower levels of renal function [53, 54], potentiating the use of MHR for risk prediction of renal dysfunction. Additionally, emerging studies have also identified a significant association between MHR and cardiovascular diseases (CVDs) [17, 18, 55, 56]. The critical role of monocytic inflammation in atherogenesis [57, 58] and the well-established beneficial effect of HDL-C on CVDs regarding its anti-inflammation and anti-atherogenic properties [14, 15, 59–61] enhanced the utility of MHR as a clinician-friendly biomarker in the risk assessment and stratification of CVDs.

### Strength and limitations of this study

Monocytic inflammation has been increasingly emphasized in the pathophysiology of T2DM in recent years. Our study, for the first time, provides epidemiological insight into the association between easily available monocytic biomarkers and T2DM incidence. Additionally, as circulating monocytes are highly dynamic and influenced by a diversity of basal behaviors and metabolic conditions [40], including eating [10] and sleeping [11], there is a need for data measured with duplicates to ensure a more rigorous and credible analysis. In this study, utilizing the repeated-measured data from the Kailuan Cohort, we applied cumulative exposure to assess chronic inflammation, thus providing more stable and reliable findings and minimizing the potential for an underestimation of the true association. Other merits of this study include the use of high-quality data from a well-designed, prospective cohort and its high maintenance of follow-up rates.

However, limitations of the current study should be addressed. First, we failed to further identify specific monocyte subsets and/or phenotypes (e.g., proinflammatory [M1] or anti-inflammatory [M2] phenotypes) to provide more detailed information on monocyte-associated inflammation in the development of T2DM. However, our results are of high importance for both clinical and epidemiological settings, providing convincing evidence of the relationship between the MHR and the risk of diabetes with widely available items in routine clinical tests. Second, we could not distinguish type 2 and type 1 diabetes in this cohort; however, there would be minimal misclassification given the advanced age of the study population relative to the younger age of onset for type 1 diabetes. Third, the current study primarily consisted of participants from an occupation-specific Han Chinese community in North China, potentially limiting the generalizability of the findings to the whole country or for other ethnicities/races. Nevertheless, the relative homogeneity of this study population in terms of diet and environmental exposures, enhances the internal validity of our findings.

## Conclusions

Our present study, for the first time, provides longitudinal epidemiological insights into the associations between monocyte-derived inflammatory markers and developing T2DM. Chronic subclinical inflammation, assessed by the CumMHR, may be an important supplement to hsCRP for a more comprehensive assessment of the inflammatory risk for T2DM. Targeted assessment and management of CumMHR in participants at low risk of diabetes, are promising ways to further reduce its occurrence.

## Supplementary Information

**Additional file 1: Table S1.** Number of participants and participations in the follow-up visits. **Table S2.** Short-term and long-term CumMHR-associated T2DM risk. **Table S3.** Stratified analysis of CumMHR-associated T2DM risks by IFG status during the exposure period. **Table S4.** Association between cumulative MHR and incident IFG among participants without IFG (8293/33722). **Table S5.** Stratified analysis of CumMHR-associated T2DM risks across CumCRP strata. **Table S6.** Stratified analysis of CumMHR-associated T2DM risks stratified by hypertensive status. **Table S7.** Stratified analysis of CumMHR-associated T2DM risks stratified by baseline overweight/obesity status. **Table S8.** Stratified analysis of CumMHR-associated T2DM risks stratified by smoking habits (yes or no). **Table S9.** Stratified analysis of cumulative MHR-associated T2DM risks stratified by baseline eGFR levels. **Table S10.** Sensitivity analyses of cumulative MHR and T2DM. **Table S11.** Sensitivity analysis of cumulative MHR and T2DM with imputed data on monocyte count, HDL-C, and hsCRP levels. **Table S12.** Association between cumulative monocyte count and incident T2DM. **Table S13.** Association between cumulative HDL-C and incident T2DM. **Table S14.** Association between baseline MHR and incident T2DM.

## Data Availability

The datasets used and/or analyzed during the current study are available from the corresponding author on reasonable request.

## Abbreviations

CIs: Confidence intervals
CKD: Chronic kidney disease
CumCRP: Cumulative high-sensitivity C-reactive protein
CumHDL: Cumulative high-density lipoprotein
CumMHR: Cumulative monocyte-to-HDL-cholesterol ratio
CumMON: Cumulative monocyte count
DBP: Diastolic blood pressure
eGFR: Estimated glomerular filtration rate
FBG: Fasting blood glucose
HDL-C: High-density lipoprotein cholesterol
HRs: Hazard ratios
hs-CRP: High-sensitivity C-reactive protein
IFG: Impaired fasting glucose
IL-1β: Interleukin-1β
IL-6: Interleukin 6
IR: Insulin resistance
LDL-C: Low-density lipoprotein cholesterol
MHR: Monocyte-to-HDL-cholesterol ratio
SBP: Systolic blood pressure
SD: Standard deviation
TC: Total cholesterol
TG: Triglyceride
TGs: Triglycerides
T2DM: Type 2 diabetes mellitus
TyG: Triglyceride-glycemia index
