# Supplementary material for: Association of cumulative monocyte to high-density lipoprotein ratio with the risk of type 2 diabetes: a prospective cohort study
Source: Cardiovasc Diabetol. 2022 Dec 3;21:268. doi: 10.1186/s12933-022-01701-7 (PMC9719154; doi:10.1186/s12933-022-01701-7)
Supplement: Supplementary file 1 — Additional file 1: Table S1. Number of participants and participations in the follow-up visits. Table S2. Short-term and long-term CumMHR-associated T2DM risk. Table S3. Stratified analysis of CumMHR-associated T2DM risks by IFG status during the exposure period. Table S4. Association between cumulative MHR and incident IFG among participants without IFG (8293/33722). Table S5. Stratified analysis of CumMHR-associated T2DM risks across CumCRP strata. Table S6. Stratified analysis of CumMHR-associated T2DM risks stratified by hypertensive status. Table S7. Stratified analysis of CumMHR-associated T2DM risks stratified by baseline overweight/obesity status. Table S8. Stratified analysis of CumMHR-associated T2DM risks stratified by smoking habits (yes or no). Table S9. Stratified analysis of cumulative MHR-associated T2DM risks stratified by baseline eGFR levels. Table S10. Sensitivity analyses of cumulative MHR and T2DM. Table S11. Sensitivity analysis of cumulative MHR and T2DM with imputed data on monocyte count, HDL-C, and hsCRP levels. Table S12. Association between cumulative monocyte count and incident T2DM. Table S13. Association between cumulative HDL-C and incident T2DM. Table S14. Association between baseline MHR and incident T2DM. [file 12933_2022_1701_MOESM1_ESM.docx]

**Additional File 1**

**Table S1. Number of Participants and participations in the follow-up visits**

| **No. of participation** | **Participants** |
| --- | --- |
| 1 | 5076 |
| 2 | 8457 |
| 3 | 12420 |
| 4 | 14860 |

**Table S2. Association between cumulative monocytes and incident type 2 diabetes**

|  | CumMON, HRs (95%CIs) | | | | *P* for trend | Per SD |
| --- | --- | --- | --- | --- | --- | --- |
|  | Quartile 1  (CumMON<0.2624) | Quartile 2  (0.2624≤CumMON<0.3394) | Quartile 3  (0.3394≤CumMON<0.4391) | Quartile 4  (CumMON≥0.4391) |  |  |
| Event/Total | 974/10204 | 1120/10204 | 1272/10202 | 1482/10203 |  |  |
| Incidence rate | 13.33 | 15.44 | 17.68 | 21.16 |  |  |
| Model (unadjusted) | Reference | 1.16 (1.06,1.26) | 1.32 (1.22,1.44) | 1.57 (1.45,1.71) | <0.001 |  |
| Model 1 | Reference | 1.10 (1.01,1.19) | 1.23 (1.13,1.34) | 1.45 (1.33,1.58) | <0.001 | 1.13 (1.10,1.16) |
| Mode1 2 | Reference | 1.09 (0.99,1.18) | 1.23 (1.13,1.34) | 1.43 (1.32,1.56) | <0.001 | 1.13 (1.09,1.15) |
| Mode1 3 | Reference | 1.06 (0.98,1.16) | 1.19 (1.09,1.29) | 1.35 (1.24,1.48) | <0.001 | 1.10 (1.07,1.13) |

Model 1: adjusted for age (continuous), sex, education, smoke, drinking status, physical activity, family history of diabetes, BMI (categorical);

Model 2: Model 1+FBG (continuous), eGFR (categorical), log(hsCRP) (continuous), dyslipidemia (yes or no), blood pressure (categorical), antihypertensives (yes or no), lipid-lowering drugs (yes or no);

Model 3: Model 2+log(leukocyte counts).

The incidence rate is per 1,000 person-years.

Abbreviation: CumMON: cumulative monocytes; BMI: body mass index; FBG: fasting blood glucose; eGFR: estimated glomerular filtration rate; hsCRP: hypersensitive C-reactive protein.

Per SD: the risk per SD increase in log (CumMON)(0.3916).

**Table S3. Association between cumulative HDL-C and incident type 2 diabetes**

|  | CumHDL, HRs (95%CIs) | | | | *P* for trend | Per SD |
| --- | --- | --- | --- | --- | --- | --- |
|  | Quartile 1  (CumHDL<1.3465) | Quartile 2  (1.3465≤CumHDL<1.5487) | Quartile 3  (1.5487≤CumHDL<1.7910) | Quartile 4  (CumHDL≥1.7910) |  |  |
| Event/Total | 1352/10204 | 1254/10202 | 1168/10204 | 1074/10203 |  |  |
| Incidence rate | 19.78 | 17.41 | 15.96 | 14.50 |  |  |
| Model (unadjusted) | Reference | 0.88 (0.80,0.95) | 0.81 (0.75,0.89) | 0.74 (0.69,0.80) | <0.001 | 0.86 (0.78,0.97) |
| Model 1 | Reference | 0.98 (0.91,1.06) | 0.96 (0.89,1.04) | 0.94 (0.87,1.02) | 0.1241 | 0.98 (0.95,1.02) |
| Model 2 | Reference | 1.02 (0.95,1.11) | 1.01 (0.93,1.09) | 1.00 (0.92,1.08) | 0.8661 | 1.00 (0.97,1.04) |

Model 1: adjusted for age (continuous), sex, education, smoke, drinking status, physical activity, family history of diabetes, BMI (categorical);

Model 2: Model 1+FBG (continuous), eGFR (categorical), log(hsCRP, continuous), dyslipidemia (yes or no), hypertension (yes or no), antihypertensives (yes or no), lipid-lowering drugs (yes or no).

The incidence rate is per 1,000 person-years.

Per SD: the risk per SD increase in log (CumHDL-C).

Abbreviation: CumHDL: cumulative high-density lipoprotein cholesterol; BMI: body mass index; FBG: fasting blood glucose; eGFR: estimated glomerular filtration rate; hsCRP: hypersensitive C-reactive protein.

**Table S4. Stratified analysis of diabetes according to cumulative MHR by IFG status during exposure period**

|  | Cum MHR, HRs (95% CIs) | | | | P for trend | Per SD |
| --- | --- | --- | --- | --- | --- | --- |
|  | Quartile 1 | Quartile 2 | Quartile 3 | Quartile 4 |  |  |
| With IFG | 2166/7091 |  |  |  |  |  |
| Event/Total | 409/1759 | 525/1821 | 535/1731 | 697/1780 |  |  |
| Incidence rate | 36.88 | 47.65 | 51.31 | 70.01 |  |  |
| Model 1 | Reference | 1.26 (1.11,1.44) | 1.34(1.17,1.52) | 1.75 (1.54,1.98) | <0.001 | 1.19 (1.14,1.24) |
| Model 2 | Reference | 1.25 (1.10,1.43) | 1.29(1.13,1.48) | 1.66 (1.46,1.88) | <0.001 | 1.15 (1.09,1.22) |
| Model 3 | Reference | 1.21 (1.06,1.38) | 1.22(1.07,1.40) | 1.51 (1.32,1.74) | <0.001 | 1.12 (1.08,1.16) |
| Without IFG | 2682/33722 |  |  |  |  |  |
| Event/Total | 481/8444 | 615/8382 | 718/8472 | 868/8424 |  |  |
| Incidence rate | 7.71 | 9.97 | 11.76 | 14.47 |  |  |
| Model 1 | Reference | 1.20 (1.06,1.35) | 1.35 (1.20,1.52) | 1.61 (1.43,1.81) | <0.001 | 1.13 (1.10,1.18) |
| Model 2 | Reference | 1.17 (1.04,1.32) | 1.29 (1.15,1.46) | 1.50 (1.33,1.69) | <0.001 | 1.11 (1.07,1.15) |
| Model 3 | Reference | 1.16 (1.02,1.31) | 1.26 (1.11,1.42) | 1.45 (1.28,1.64) | <0.001 | 1.09 (1.05,1.13) |

P-interaction: CumMHR quartiles × IFG status (yes or no) = 0.6767.

Model 1: adjusted for age (continuous), sex, education, smoke, drinking status, physical activity, family history of diabetes, BMI (categorical);

Model 2: Model 1+ FBG (continuous), dyslipidemia (yes or no), blood pressure (categorical), eGFR(categorical), anti-hypertensives (yes or no), lipid-lowering drugs (yes or no), log(hsCRP, continuous);

Model 3: Model 2+ log(leukocyte counts).

The incidence rate is per 1,000 person-years.

Per SD: the risk per SD increase in log (CumMHR) (0.4594).

Abbreviation: CumMHR: cumulative monocyte to high-density lipoprotein cholesterol ratio; BMI: body mass index; FBG: fasting blood glucose; eGFR: estimated glomerular filtration rate; hsCRP: hypersensitive C-reactive protein; IFG: impaired fasting glucose.

**Table S5.** **Stratified analysis of diabetes according to cumulative MHR by CumCRP level**

|  | CumMHR, HRs (95% CIs) | | | | *P* for trend | Per SD |
| --- | --- | --- | --- | --- | --- | --- |
|  | Quartile 1 | Quartile 2 | Quartile 3 | Quartile 4 |  |  |
| CumCRP<1 mg/L | 1049/12920 |  |  |  |  |  |
| Event/Total | 259/4365 | 268/3378 | 256/2909 | 266/2268 |  |  |
| Incidence rate | 8.12 | 10.87 | 12.39 | 16.89 |  |  |
| Model 1 | Reference | 1.25 (1.05,1.49) | 1.37 (1.15,1.63) | 1.85 (1.54,2.21) | <0.0001 | 1.20 (1.12,1.28) |
| Model 2 | Reference | 1.26 (1.05,1.49) | 1.37 (1.14,1.64) | 1.71 (1.43,2.06) | <0.0001 | 1.15 (1.08,1.23) |
| Model 3 | Reference | 1.22 (1.02,1.45) | 1.30 (1.08,1.57) | 1.59 (1.31,1.92) | <0.0001 | 1.11 (1.04,1.20) |
| 1≤CumCRP<3 mg/L | 2254/17412 |  |  |  |  |  |
| Event/Total | 437/4146 | 566/4418 | 553/4388 | 698/4460 |  |  |
| Incidence rate | 14.73 | 18.15 | 17.98 | 22.79 |  |  |
| Model 1 | Reference | 1.20 (1.05,1.36) | 1.17 (1.03,1.33) | 1.47 (1.29,1.66) | <0.0001 | 1.10 (1.05,1.15) |
| Model 2 | Reference | 1.20 (1.05,1.36) | 1.15 (1.01,1.31) | 1.48 (1.30,1.68) | <0.0001 | 1.10 1.06,1.15) |
| Model 3 | Reference | 1.17 (1.03,1.33) | 1.12 (0.98,1.28) | 1.40 (1.22,1.61) | <0.0001 | 1.08 (1.03,1.13) |
| 3≤CumCRP<10 mg/L | 1332/8898 |  |  |  |  |  |
| Event/Total | 169/1496 | 274/2079 | 398/2467 | 491/2856 |  |  |
| Incidence rate | 16.19 | 18.95 | 23.52 | 25.30 |  |  |
| Model 1 | Reference | 1.09 (0.90,1.32) | 1.32 (1.10,1.58) | 1.38 (1.15,1.66) | <0.0001 | 1.13 (1.07,1.19) |
| Model 2 | Reference | 1.10 (0.91,1.34) | 1.37 (1.14,1.65) | 1.47 (1.22,1.76) | <0.0001 | 1.15 (1.10,1.21) |
| Model 3 | Reference | 1.09 (0.90,1.33) | 1.34 (1.11,1.61) | 1.41(1.16,1.71) | <0.0001 | 1.15 (1.09,1.21) |
| CumCRP≥10 mg/L | 213/1583 |  |  |  |  |  |
| Event/Total | 25/196 | 32/328 | 46/439 | 110/620 |  |  |
| Incidence rate | 17.18 | 13.38 | 14.53 | 26.49 |  |  |
| Model 1 | Reference | 0.77 (0.45,1.29) | 0.83 (0.51,1.35) | 1.49 (0.96,2.30) | 0.0722 | 1.10 (0.99,1.22) |
| Model 2 | Reference | 0.60 (0.35,1.02) | 0.58 (0.35,0.95) | 1.04 (0.65,1.65) | 0.0864 | 1.09 (0.99,1.21) |
| Model 3 | Reference | 0.60 (0.35,1.03) | 0.58 (0.35,0.97) | 1.08 (0.65,1.73) | 0.1001 | 1.10 (0.98,1.25) |

Model 1: adjusted for age (continuous), sex, education, smoke, drinking status, physical activity, family history of diabetes, BMI (categorical);

Model 2: Model 1+ FBG (continuous), dyslipidemia (yes or no), blood pressure (categorical), eGFR(categorical), anti-hypertensives (yes or no), anti-lipidemia (yes or no);

Model 3: Model 2+ log(leukocyte counts).

The incidence rate is per 1,000 person-years.

P-interaction: CumMHR quartiles × CumCRP thresholds (1, 3, 10 mg/L) = 0.0146.

Per SD: the risk per SD increase in log (CumMHR) (0.4594).

Abbreviation: CumMHR: cumulative monocyte to high-density lipoprotein cholesterol ratio; CumCRP: cumulative hypersensitive C-reactive protein; BMI: body mass index; FBG: fasting blood glucose; eGFR: estimated glomerular filtration rate.

**Table S6.** **Stratified analysis of diabetes and cumulative MHR by hypertensive status**

|  | CumMHR, HRs (95% CIs) | | | | *P* for trend | Per SD |
| --- | --- | --- | --- | --- | --- | --- |
|  | Quartile 1 | Quartile 2 | Quartile 3 | Quartile 4 |  |  |
| Hypertension | 3072/19733 |  |  |  |  |  |
| Event/Total | 547/4373 | 718/4865 | 804/5101 | 1003/5394 |  |  |
| Incidence rate | 18.38 | 21.80 | 23.60 | 28.44 |  |  |
| Model 1 | Reference | 1.13 (1.01,1.26) | 1.18 (1.06,1.32) | 1.38 (1.24,1.54) | <0.001 | 1.10 (1.06,1.14) |
| Model 2 | Reference | 1.16 (1.03,1.30) | 1.20 (1.08,1.34) | 1.42 (1.27,1.58) | <0.001 | 1.11 (1.07,1.15) |
| Model 3 | Reference | 1.11 (0.99,1.25) | 1.14 (1.02,1.28) | 1.30 (1.15,1.46) | <0.001 | 1.10 (1.06,1.14) |
| Non-hypertension | 1776/21080 |  |  |  |  |  |
| Event/Total | 343/5830 | 422/5338 | 449/5102 | 562/4810 |  |  |
| Incidence rate | 7.85 | 10.62 | 11.99 | 16.21 |  |  |
| Model 1 | Reference | 1.21 (1.05,1.40) | 1.28 (1.10,1.49) | 1.58 (1.35,1.84) | <0.001 | 1.18 (1.13,1.23) |
| Model 2 | Reference | 1.26 (1.09,1.46) | 1.40 (1.21,1.62) | 1.84 (1.59,2.12) | <0.001 | 1.18 (1.13,1.23) |
| Model 3 | Reference | 1.21 (1.05,1.40) | 1.28 (1.10,1.49) | 1.58 (1.35,1.84) | <0.001 | 1.13 (1.07,1.18) |

Model 1: adjusted for age (continuous), sex, education, smoke, drinking status, physical activity, family history of diabetes, BMI (categorical);

Model 2: Model 1+ FBG (continuous), eGFR (categorical), log(hsCRP, continuous) , dyslipidemia (yes or no), antihypertensives (yes or no), lipid-lowering drugs (yes or no);

Model 3: Model 2 +log(leukocyte counts).

The incidence rate is per 1,000 person-years.

P-interaction: CumMHR quartiles × hypertension history (yes or no) = 0.0193.

Per SD: the risk per SD increase in log (CumMHR) (0.4594).

Abbreviations as ESM Table 5.

**Table S7. Stratified analysis of diabetes and cumulative MHR stratified by baseline overweight status**

|  | CumMHR, HRs (95% CIs) | | | | *P* for trend | Per SD |
| --- | --- | --- | --- | --- | --- | --- |
|  | Quartile 1 | Quartile 2 | Quartile 3 | Quartile 4 |  |  |
| Overweight | 3184/19683 |  |  |  |  |  |
| Event/Total | 495/3848 | 736/4713 | 854/5309 | 1099/5813 |  |  |
| Incidence rate | 18.92 | 21.85 | 23.64 | 28.47 |  |  |
| Model 1 | Reference | 1.25 (1.11,1.40) | 1.32 (1.18,1.47) | 1.61 (1.44,1.79) | <0.001 | 1.12 (1.08,1.16) |
| Model 2 | Reference | 1.22 (1.09,1.37) | 1.29 (1.16,1.45) | 1.55 (1.39,1.73) | <0.001 | 1.11 (1.07,1.15) |
| Model 3 | Reference | 1.20 (1.07,1.35) | 1.25 (1.12,1.41) | 1.47 (1.31,1.65) | <0.001 | 1.10 (1.06,1.14) |
| Non-overweight | 1664/21130 |  |  |  |  |  |
| Event/Total | 395/6355 | 404/5490 | 399/4894 | 466/4391 |  |  |
| Incidence rate | 8.51 | 10.13 | 11.39 | 15.15 |  |  |
| Model 1 | Reference | 1.18 (1.02,1.36) | 1.34 (1.16,1.54) | 1.79 (1.56,2.06) | <0.001 | 1.18 (1.13,1.23) |
| Model 2 | Reference | 1.18 (1.03,1.36) | 1.29 (1.11,1.48) | 1.71 (1.49,1.98) | <0.001 | 1.17 (1.12,1.22) |
| Model 3 | Reference | 1.18 (1.02,1.36) | 1.28 (1.11,1.48) | 1.70 (1.47,1.98) | <0.001 | 1.13 (1.07,1.18) |

Model 1: adjusted for age (continuous), sex, education, smoke, drinking status, physical activity, family history of diabetes.

Model 2: Model 1 + FBG (continuous), eGFR (categorical), log(hsCRP) (continuous), dyslipidemia (yes or no), blood pressure (categorical), anti-hypertensives (yes or no), lipid-lowering drugs (yes or no);

Model 3: Model 2+ log (leukocyte counts).

P-interaction: CumMHR quartiles × overweight status (yes or no) = 0.0384.

Per SD: the risk per SD increase in log (CumMHR) (0.4594).

Abbreviations as ESM Table 5.

**Table S8. Sensitivity analysis of cumulative MHR and type 2 diabetes**

|  | CumMHR, HRs (95%CIs) | | | |  |  |
| --- | --- | --- | --- | --- | --- | --- |
|  | Quartile 1 | Quartile 2 | Quartile 3 | Quartile 4 | *P* for trend | P for SD |
| Excluded acute infection (whichever hsCRP≥10mg/L during the exposure period) (4285/36744) | | | | | | |
| Model 1 | Reference | 1.22 (1.11,1.33) | 1.30 (1.19,1.43) | 1.60 (1.46,1.76) | <0.001 | 1.14 (1.11,1.18) |
| Model 2 | Reference | 1.22 (1.11,1.34) | 1.31 (1.19,1.43) | 1.61 (1.46,1.76) | <0.001 | 1.14 (1.11,1.18) |
| Model 3 | Reference | 1.19 (1.09,1.31) | 1.26 (1.14,1.38) | 1.51 (1.37,1.66) | <0.001 | 1.12 (1.08,1.15) |
| Excluded use of statin (4804/40583) | | | | | | |
| Model 1 | Reference | 1.20 (1.10,1.32) | 1.29 (1.18,1.41) | 1.60 (1.47,1.75) | <0.001 | 1.14 (1.11,1.18) |
| Model 2 | Reference | 1.21 (1.11,1.32) | 1.29 (1.18,1.40) | 1.60 (1.47,1.75) | <0.001 | 1.14 (1.11,1.18) |
| Model 3 | Reference | 1.19 (1.09,1.30) | 1.25 (1.14,1.37) | 1.52 (1.38,1.67) | <0.001 | 1.12 (1.09,1.15) |
| Excluded participants with baseline CVD (4498/38577) | | | | | | |
| Model 1 | Reference | 1.18 (1.08,1.29) | 1.31 (1.19,1.43) | 1.60 (1.46,1.75) | <0.001 | 1.15 (1.11,1.18) |
| Model 2 | Reference | 1.19 (1.08,1.30) | 1.31 (1.19,1.43) | 1.59 (1.45,1.74) | <0.001 | 1.14 (1.11,1.18) |
| Model 3 | Reference | 1.17 (1.06,1.28) | 1.26 (1.15,1.38) | 1.50 (1.36,1.65) | <0.001 | 1.12 (1.08,1.15) |
| Excluded diabetes occurred within the follow-up visit (3409/39374) | | | | | | |
| Model 1 | Reference | 1.23 (1.11,1.37) | 1.33 (1.20,1.48) | 1.62 (1.46,1.80) | <0.001 | 1.13 (1.09,1.17) |
| Model 2 | Reference | 1.24 (1.12,1.38) | 1.34 (1.20,1.48) | 1.64 (1.48,1.82) | <0.001 | 1.14 (1.10,1.17) |
| Model 3 | Reference | 1.22 (1.10,1.36) | 1.29 (1.16,1.44) | 1.54 (1.32,1.73) | <0.001 | 1.11 (1.07,1.15) |
| Excluded missing data (4821/40592) | | | | | | |
| Model 1 | Reference | 1.20 (1.10,1.31) | 1.29 (1.18,1.41) | 1.58 (1.45,1.73) | <0.001 | 1.14 (1.11,1.17) |
| Model 2 | Reference | 1.20 (1.10,1.31) | 1.28 (1.17,1.40) | 1.57 (1.44,1.72) | <0.001 | 1.14 (1.11,1.17) |
| Model 3 | Reference | 1.18 (1.08,1.29) | 1.24 (1.14,1.36) | 1.50 (1.36,1.64) | <0.001 | 1.11 (1.08,1.15) |

Model 1: adjusted for age (continuous), sex, education, smoke, drinking status, physical activity, family history of diabetes, BMI (categorical);

Model 2: Model 1 + FBG (continuous), eGFR (categorical), log(hsCRP)(continuous), dyslipidemia (yes or no), blood pressure (categorical), antihypertensives (yes or no), lipid-lowering drugs (yes or no).

Model 3: Model 2 + log (leukocyte counts).

Per SD: the risk per SD increase in log (CumMHR) (0.4594).

Abbreviations as ESM Table 5.

**Table S9. Association between baseline MHR and incident type 2 diabetes**

|  | BasMHR, HRs (95% CIs) | | | | *P* for trend | Per SD |
| --- | --- | --- | --- | --- | --- | --- |
|  | Quartile 1  (BasMHR<0.1449) | Quartile 2  (0.1449≤BasMHR<0.2162) | Quartile 3  (0.2162≤BasMHR<0.32) | Quartile 4  (0.2162≤BasMHR<0.32) |  |  |
| Event/Total | 959/10109 | 1070/10284 | 1313/10119 | 1506/10301 |  |  |
| Incidence rate | 13.01 | 14.69 | 18.72 | 21.24 |  |  |
| Model(unadjusted) | Reference | 1.12 (1.02,1.22) | 1.42 (1.30,1.54) | 1.60 (1.47,1.73) | <0.0001 | 1.12 (1.10,1.14) |
| Model 1 | Reference | 1.04 (0.96,1.14) | 1.24 (1.14,1.35) | 1.36 (1.25,1.47) | <0.0001 | 1.08 (1.05,1.10) |
| Mode12 | Reference | 1.02 (0.93,1.11) | 1.23 (1.13,1.34) | 1.31 (1.20,1.43) | <0.0001 | 1.06 (1.03,1.09) |
| Mode13 | Reference | 0.99 (0.91,1.08) | 1.17 (1.07,1.28) | 1.22 (1.11,1.33) | <0.0001 | 1.03 (1.01,1.06) |

Model 1: adjusted for age (continuous), sex, education, smoke, drinking status, physical activity, family history of diabetes, BMI (categorical);

Model 2: Model 1 + FBG (continuous), eGFR (categorical), log(hsCRP, continuous), dyslipidemia (yes or no), blood pressure (categorical), antihypertensives (yes or no), lipid-lowering drugs (yes or no);

Model 3: Model 2 + log(leukocyte counts) (continuous).

Per SD: the risk per SD increase in log (BasMHR).

Abbreviations: BasMHR: baseline monocyte to high-density lipoprotein cholesterol ratio; others as ESM Table 5.
